# Fuzzy masks: boosting radiomic reliability in head and neck tumors amid delineation uncertainty^☆^

**DOI:** 10.1016/j.phro.2026.100947

**Published:** 2026-03-15

**Authors:** Jin Cao, Jiang Zhang, Xinzhi Teng, Xinyu Zhang, Saikit Lam, Ta Zhou, Yuanpeng Zhang, Jing Cai

**Affiliations:** a Department of Health Technology and Informatics, The Hong Kong Polytechnic University, Hong Kong SAR, China; b Department of Biomedical Engineering, The Hong Kong Polytechnic University, Hong Kong SAR, China; c School of Computing, Jiangsu University of Science and Technology, Zhenjiang 212100, China; d Department of Medical Informatics, Nantong University, Nantong 226007, China; e The Hong Kong Polytechnic University Shenzhen Research Institute, Shenzhen 518057, China

**Keywords:** Radiomics, Head and neck cancer, Computed tomography, Reliability, Fuzzy mask

## Abstract

**Background and purpose:**

The clinical utility of radiomics in head-and-neck (H&N) cancer is hindered by poor reliability caused by delineation uncertainties from the use of binary mask (BinMask). This study introduced a fuzzy mask (FuzzMask) approach to enhance the reliability of computed tomography (CT)-based radiomics for precision prognosis.

**Materials and methods:**

This retrospective study included 2,539 H&N cancer patients (855 laryngeal cancer (LC), 1,336 oropharyngeal cancer (OPC), 348 nasopharyngeal carcinoma (NPC)). Delineation uncertainty was simulated via perturbation techniques. Radiomic features (RFs) were extracted using BinMask and FuzzMask, respectively. The evaluation focused on feature reliability and relevance via the intraclass correlation coefficient (ICC) and hierarchical clustering. In addition, the predictive performance and output reliability of penalized Cox’s proportional hazard models were assessed using the concordance index (C-index) and ICC, respectively.

**Results:**

The FuzzMask improved feature reliability, yielding 21, 29, and 5 additional reliable features for LC, OPC, and NPC cancers, respectively, compared to BinMask. The FuzzMask also reduced feature redundancy, generating up to 70 more clusters in hierarchical clustering, particularly for smaller tumors in complex peritumoral environments although showed marginal improvements in predictive performance (C-index: +0.1% for OPC, +0.4% for NPC, *p* > 0.05). However, model reliability was enhanced by FuzzMask, with ICC values increasing by 0.024, 0.022, and 0.007 for LC, OPC, and NPC, respectively, compared to BinMask (*p* ≥ 0.05).

**Conclusions:**

The proposed FuzzMask technique significantly improved feature reliability and model robustness against delineation uncertainty, offering greater trustworthiness for clinical translation, although predictive accuracy remains unaffected.

## Highlights

• Fuzzy mask models tumor contour uncertainty via gradient transitions.
• The method yields up to 29 more reliable features than binary masks.
• Gradient weighting produces 2% more independent feature clusters.
• Intraclass correlation coefficient of the predictive outputs of model up to 0.99.
• Intensity equalization mechanisms drive the observed reliability gains.

## Graphical abstract

**Figure:**
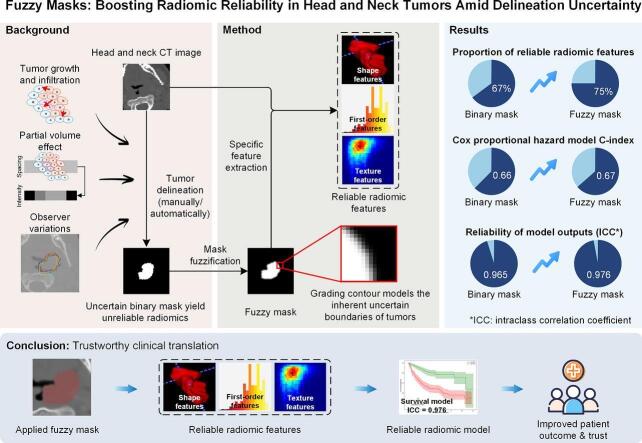

## 1 Introduction

Radiomics, a method designed to extract high-dimensional image features (called radiomic features, (RFs)) from regions-of-interest, has been widely adopted for developing image biomarkers [1]. These biomarkers enable accurate disease diagnosis, prognosis prediction, and assessment of treatment response [2], [3], [4], [5]. A key requirement for the clinical translation of radiomics is ensuring their reliability, which refers to the consistency of RFs and the output of radiomics models despite variations in tumor delineation [6], [7], [8]. However, achieving such reliability amid delineation uncertainties remains a significant challenge.

Tumor delineation/segmentation is typically performed manually by a radiologist, a task that is tedious, time-consuming, and prone to error due to factors such as human fatigue, an overabundance of slices per patient, and intra-observer variability. Such manual operations often lead to inaccurate delineation, potentially resulting in unstable and unreliable outcomes in radiomics-based analysis [9], [10], [11].

Deep learning-based segmentation methods can effectively improve the consistency of tumor segmentation [12], [13], thereby enhancing the reliability of radiomic analysis. Yet model bias and performance variations still cause radiomics inconsistencies [8].

Regardless of whether the mask is segmented manually or automatically, the primary delineation uncertainty arises from the need for an accurate and clear binary mask [14]. The term “binary” implies that each voxel is “clearly” classified as either a member of the tumor or not. However, issues such as the partial volume effect (PVE) or beam hardening effects in computed tomography (CT) lead to inconsistent intensities within tissues [15]. Additionally, microscopic invasion of tumor cells results in unclear tumor boundaries in imaging [11]. Consequently, obtaining a clear binary mask in the presence of uncertain tumor boundaries, coupled with inter- and intra-observer variability in the delineation process, contributes to delineation uncertainties in radiomics. Therefore, studies are transitioning from binary mask (BinMask) to fuzzy mask (FuzzMask) to address the boundary uncertainty.

This transition is justified by FuzzMask’s ability to model uncertainty by allowing pixels to have partial object membership. Its efficacy has been empirically validated in multiple segmentation studies, which demonstrated superior accuracy and robustness compared to conventional binary methods [15], [16], [17], [18], [19], [20]. In radiomics, Papp et al. [14], [21] introduced a FuzzMask that was directly applied to extract RFs from positron emission tomography (PET) images and achieved an improved predictive performance of radiomics models. However, despite these advantages, the application of FuzzMask has not been fully explored for CT imaging in head-and-neck (H&N) cancer.

While tumor boundaries on CT images are generally clearer than on PET, the complexity of tissues and organs in H&N presents unique challenges. These include varying image intensities near tumor boundaries, leading to complex shapes and making accurate delineation difficult. Consequently, this variability significantly impacts the reliability of radiomics analysis.

To address these challenges, this study aimed to apply FuzzMask for extracting reliable RFs from CT images in H&N cancer, potentially enhancing both the performance and reliability of radiomics models.

## 2 Materials and methods

**Fig. 1:**
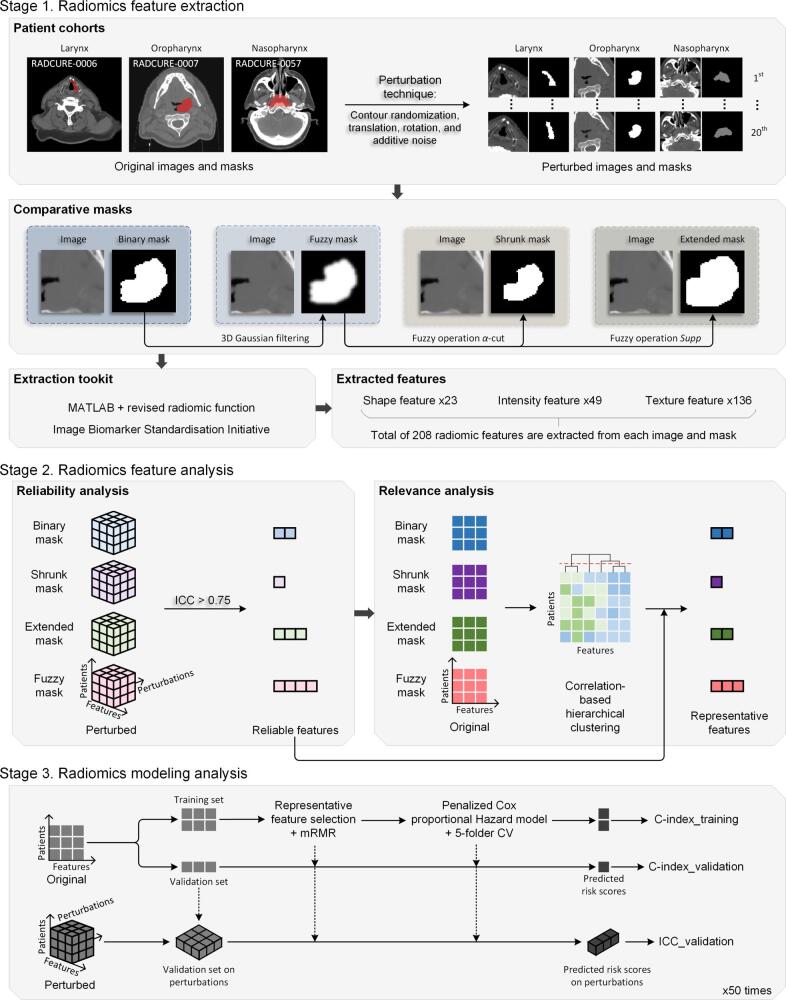
The overall workflow of this study. Stage 3 applies to conventional binary mask, extended binary mask, and fuzzy mask, with only one demonstrated here for clarity. Dotted line arrows in Stage 3 indicate that the same operation is repeated using identical parameters.

The overall workflow of this retrospective study is illustrated in Fig. 1. It comprises three stages: RF extraction, analysis, and modeling, which are detailed in the subsections.

### 2.1 Patient cohorts

**Fig. 2:**
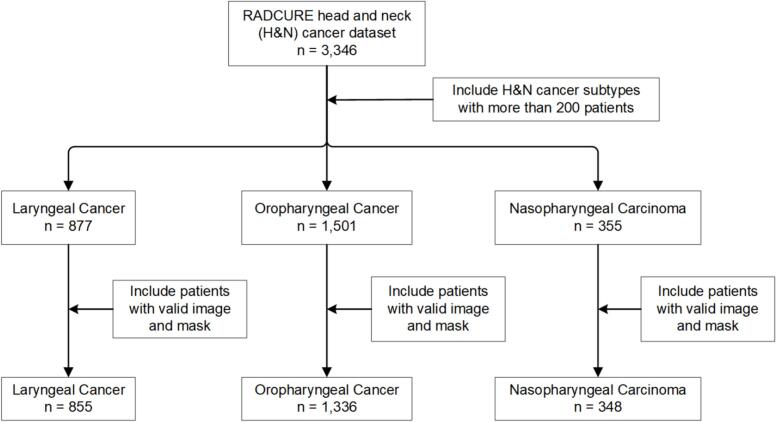
Patient inclusion flowchart based on the RADCURE dataset [22]. Three H&N cancer subtypes (LC, OPC, NPC) were selected based on a sample size threshold of >200 to ensure data sufficiency. The final inclusion required the concurrent availability of valid paired CT images and segmentation masks.

The publicly available RADCURE dataset [22] comprises 3,346 H&N CT image volumes. This dataset includes primary gross-tumor-volume (GTVp), relevant gross lymph nodes, and organs-at-risk (OAR), delineated and reviewed for radiotherapy planning. However, as this study specifically focuses on tumor-derived radiomics, only the GTVp contours were utilized for the subsequent analysis. This study examined three subtypes of H&N cancers from the dataset: laryngeal cancer (LC), oropharyngeal cancer (OPC), and nasopharyngeal carcinoma (NPC). The patient inclusion diagram is illustrated in Fig. 2, and the characteristics of the included cohorts are summarized in Table 1.

**Table 1:** Selected demographic and clinical characteristics of the three study cohorts derived from the RADCURE dataset [22]. Continuous variables are presented as Mean ± Standard Deviation (SD), while categorical variables are expressed as counts (percentages).

Characteristics		LC (n = 855)	OPC (n = 1,336)	NPC (n = 348)
Age (years)	Mean ± SD	66.73 ± 11.13	61.5 ± 9.82	52.87 ± 12.7
Gender	Male (%)	733 (85.7%)	1,079 (80.8%)	249 (71.55%)
	Female (%)	122 (14.3%)	257 (19.2%)	99 (28.45%)
Smoking	Smoker (%)	743 (86.9%)	982 (73.5%)	141 (40.52%)
	Non-smoker (%)	112 (13.1%)	354 (26.5%)	207 (59.48%)
Tumor volume (voxels)	Mean	9,583	32,498	37,790
Stage	0 (%)	39 (4.5%)	0 (0%)	0 (0%)
	I (%)	268 (31.4%)	19 (1.4%)	13 (3.7%)
	II (%)	214 (25.0%)	72 (5.4%)	41 (11.8%)
	III (%)	170 (19.9%)	168 (12.6%)	162 (46.6%)
	IV (%)	164 (19.2%)	1077 (80.6%)	132 (37.9%)
Survival status	Alive (%)	586 (68.5%)	909 (68.0%)	295 (84.8%)
	Dead (%)	269 (31.5%)	427 (32.0%)	53 (15.2%)
Follow-up duration (years)	Mean ± SD	3.82 ± 2.43	2.51 ± 2.70	5.35 ± 3.16

### 2.2 Fuzzy mask and comparative masks

Four masks were evaluated in this experiment: conventional BinMask, shrunk binary mask (ShrMask), extended binary mask (ExtMask), and FuzzMask, as shown in Stage 1 of Fig. 1. The BinMask served as the baseline. The inclusion or exclusion of uncertain boundary regions can enhance radiomics reliability [14], prompting the use of ShrMask and ExtMask as control masks.

The FuzzMask was generated by applying Gaussian filtering to the BinMask, as described in prior studies on FuzzMask [14], [21]. The degree of fuzziness was determined by the standard deviation σ of the three-dimensional Gaussian filter applied to the volumetric CT data. In this study, we employed patient-specific pixel size (referred to as *u*, i.e., σ=u) as a surrogate for full-width half-maximum, which is used in [14].

The ShrMask and ExtMask were derived from FuzzMask using α-cut and *Supp* techniques, respectively (detailed in Section A of the Supplementary Material). Specifically, the ShrMask comprised voxels with a value of 1 in FuzzMask, while ExtMask included all non-zero voxels in FuzzMask. For small tumors (<100 voxels), all FuzzMask values could fall below 1, preventing ShrMask generation and reducing its patient cohort. To ensure a fair comparison and reduce bias, patients were stratified by tumor volume for RF reliability and relevance analysis.

### 2.3 Radiomics feature extraction

Mask preprocessing and feature extraction procedures were implemented using MATLAB R2024b. The RF extraction process, developed into a toolkit of MATLAB R2023b following the image biomarker standardisation initiative (IBSI) guidelines, was further developed by the inclusion of a graphical user interface for RF extraction using FuzzMask, available via GitHub.^1^ A detailed list of extracted RFs is provided on the official MATLAB website, and the preprocessing and extraction process using FuzzMask is further elaborated in Section B and Section C of the Supplementary Material.

### 2.4 Radiomics feature analysis

The reliability of RFs refers to their consistency under delineation uncertainties. In this study, a perturbation technique was employed to simulate such variations. This method utilizes contour randomization, translation, rotation, and additive noise [23] to simulate delineation uncertainties. We created 20 perturbed images and masks with randomly selected perturbation parameters (the parameter values are listed in Table S1 of the Supplementary Material).

The quantitative evaluation of RF reliability was conducted using the intraclass correlation coefficient (ICC) based on a two-way random-effects model for consistency (ICC(C,1)) [24], [25]. Reliability values were categorized as poor (<0.5), moderate (0.5–0.75), good (0.75–0.9), or excellent (>0.9) reliability [26], [27].

To compare the performance of distinct masks across tumors of varying sizes, patients were stratified by tumor voxel volume in 5k increments to facilitate reliability analysis of mask-derived features per tumor-size subgroup. Additionally, the reliability of FuzzMask features was assessed across fuzziness levels corresponding to distinct standard deviations (σ= 0.5 u, σ= 1 u, and σ= 1.5 u).

To analyse the relevance of RFs extracted from four comparative masks, a hierarchical clustering method was employed in MATLAB using the *linkage* function with ‘correlation’ as the distance metric. Clusters were determined through a threshold of 0.25, meaning that RFs whose distance was smaller than 0.25 were aggregated into the same cluster. Consequently, a higher cluster count signifies lower relevance of the extracted features.

### 2.5 Modeling analysis

As shown in Stage 3 of Fig. 1, the modeling process was repeated 50 times to eliminate the evaluation bias caused by the selection of the patients in the validation set. In each iteration, the patients were divided into training and validation sets using a 7:3 ratio. During the training process, 5-fold cross-validation was employed to determine the optimal feature numbers and model parameters. Prior to training the penalized Cox’s proportional hazard model [28], [29], feature candidates were identified through reliability analysis, relevance analysis, and ranking using a max-relevance and min-redundancy algorithm. After training the survival model with those optimal features and parameters on the entire training set, it was evaluated on the validation set to obtain the prediction concordance index (C-index). The resulting 50 C-index values for each mask were compared using a Wilcoxon Rank Sum Test to assess statistical significance. Furthermore, to mitigate the potential impact of sample imbalance on model evaluation, the mean time-dependent area under the curve (AUC) for predicting 1-, 3-, and 5-year survival status was calculated as an additional performance metric.

To quantify the pre-trained model’s reliability against delineation uncertainty, we generated 20 sets of risk scores by applying the model to 20 perturbed validation datasets. The ICC(C,1) [26] was calculated across these scores to measure output consistency, as shown in Stage 3 of Fig. 1.

## 3 Results

**Fig. 3:**
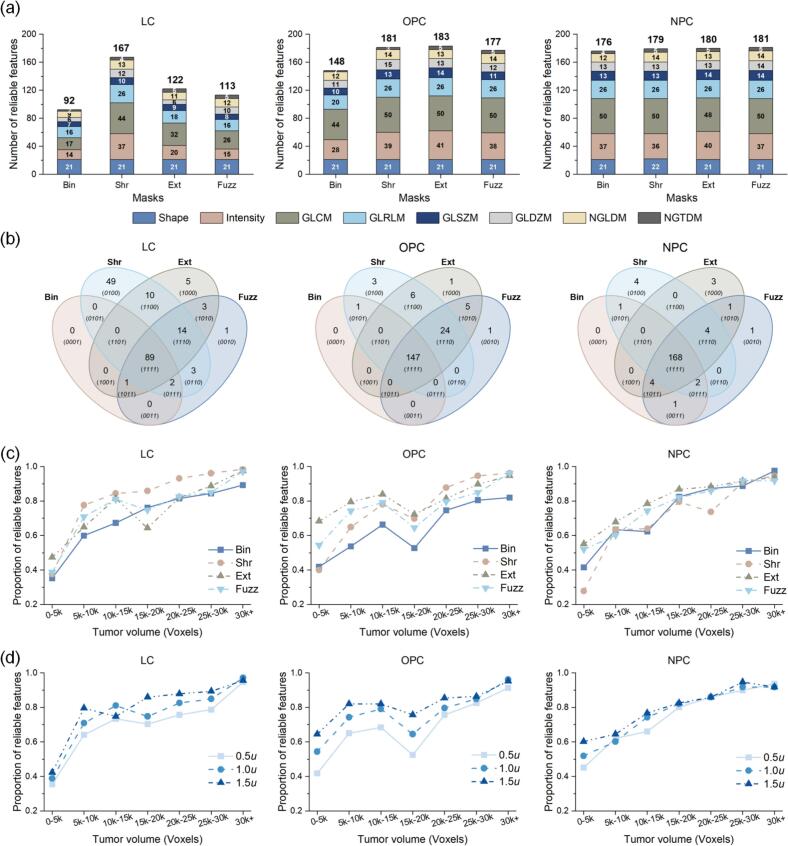
Reliability analysis results of RFs. (a) Number of reliable features (ICC ≥ 0.75) extracted using different masks. Bold numbers above stacked bars indicate the total number of reliable features, and numbers within bars denote contributions from each feature category. (b) The Venn diagrams illustrate shared and unique reliable features among different masks. (c) Proportion of reliable features stratified by tumor size. (d) Proportion of reliable features across fuzziness levels.

As illustrated in Fig. 3(a), the features extracted by FuzzMask demonstrated enhanced reliability, yielding 21, 29, and 5 additional reliable features for LC, OPC, and NPC, respectively. Comparatively, increases of 75, 33, and 3 features were observed with ExtMask, while ShrMask yielded increases of 30, 35, and 4 features for the same groups. As indicated by the stacked colored bars, these newly identified reliable features were predominantly intensity and gray-level co-occurrence matrix (GLCM)-derived features.

The Venn diagram in Fig. 3(b) illustrates that, compared with BinMask, the primary contributor to FuzzMask’s enhancement of feature reliability was the same as for ShrMask and ExtMask (category (1110) in the diagram), with specific feature names detailed in Tables S2 and S4 of the Supplementary Material, while FuzzMask itself only rendered one feature (ngldm_DependenceCountPercentage3D) reliable (category (0010)).

Fig. 3(c) and (d) confirmed that in OPC and NPC patients with intricate peritumoral environments, small tumors yielded significantly divergent proportions of reliable features across mask types and fuzziness levels. For the LC cohort, tumors with >5 k voxels aligned with OPC and NPC results; yet, no significant performance differences emerged across mask methods for small LC tumors (≤5k voxels).

**Fig. 4:**
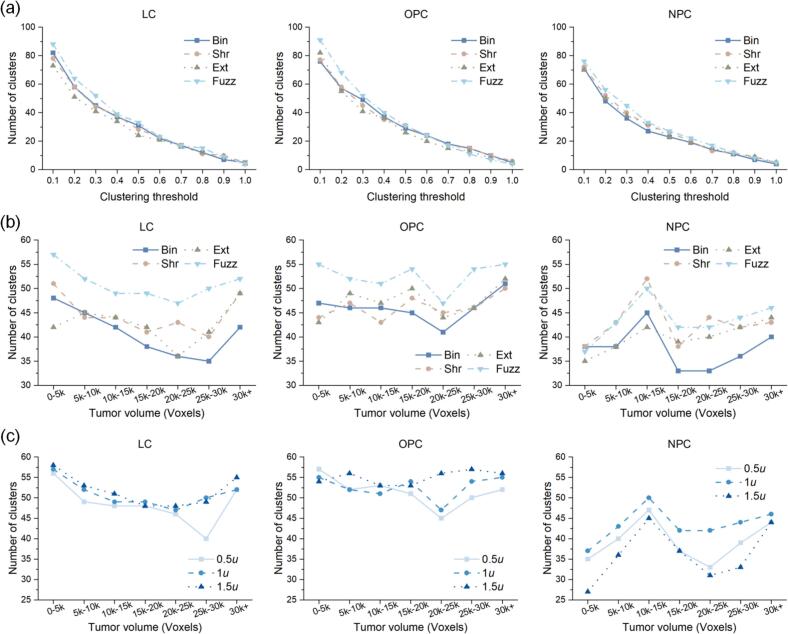
Relevance analysis results of RFs. (a) Number of feature clusters identified by hierarchical clustering under various clustering thresholds for RFs derived from four comparative masks. (b) Number of clusters stratified by tumor size at a clustering threshold of 0.25. (c) Number of clusters across different fuzzification parameters (fuzziness increased from 0.5*u* to 1.5*u*) and tumor size at a clustering threshold of 0.25.

Regarding the relevance of RFs, as illustrated in Fig. 4(a), the FuzzMask yielded a higher number of clusters at thresholds below 0.5 across the LC, OPC, and NPC cohorts compared to other methods. The results in Fig. 4(b) indicated that FuzzMask consistently produced the most clusters for the LC and OPC cohorts across various tumor sizes. For the NPC cohort, where it was not the top performer, the FuzzMask significantly outperformed the traditional BinMask. Regarding fuzziness levels, Fig. 4(c) reveals that while the LC cohort was insensitive to fuzziness parameters (σ= 0.5*u*, σ= 1*u*, σ= 1.5*u*), the NPC cohort exhibited a clear performance divergence, where higher fuzziness (σ= 1.5*u*) results in the fewest clusters.

**Fig. 5:**
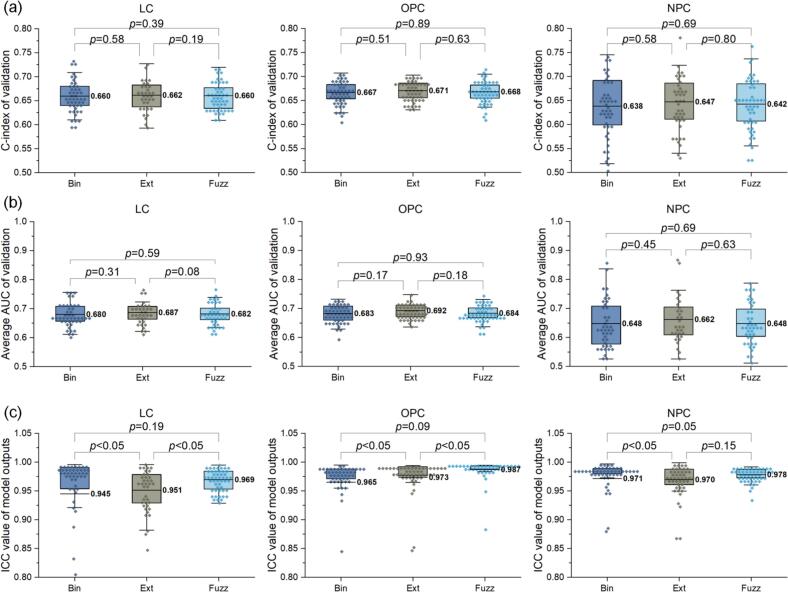
Predictive performance and reliability of penalized Cox proportional hazards models, illustrated by box plots with mean, the 25% and 75% percentiles, and data points within the box. (a) Comparison of validation C-indices. (b) Average AUCs of 1-, 3-, and 5-year survival status predictions. (c) ICC values of model predictions based on perturbated validation data.

In terms of the predictive performance of the radiomics models, as presented in Fig. 5(a), the FuzzMask outperformed BinMask by 0.1% and 0.4% in terms of C-index for OPC and NPC, respectively, with no observable change for LC. Nevertheless, these minor increments were statistically non-significant (*p* > 0.05). Higher performance gains were achieved by ExtMask across the LC (0.2%), OPC (0.4%), and NPC (0.9%) cohorts, yet without statistical difference (*p* > 0.05). In parallel, the AUC analysis in Fig. 5(b) yielded results consistent with the C-index-based evaluation.

Finally, focusing on the reliability of the radiomics models, as illustrated in Fig. 5(c), the average ICC values for model predictions utilizing FuzzMask were higher than those of BinMask by 0.024, 0.022, and 0.007 for the LC, OPC, and NPC cohorts, respectively. In contrast, the ExtMask-based model exhibited differences of 0.006, 0.008, and −0.001 relative to BinMask for the same groups.

## 4 Discussion

This study investigated the utility of FuzzMask in H&N CT radiomics, focusing on its impact on feature reliability, relevance, and predictive modeling performance. Our evaluation revealed that FuzzMask significantly enhanced the reliability and reduced the redundancy of RFs compared to conventional BinMask, with this advantage being most pronounced in small tumors with complex peritumoral anatomy. While these feature-level refinements did not yield statistically significant gains in discriminative power (C-index and AUC), the reliability of predictive outputs of the model against delineation uncertainty was substantially improved by FuzzMask.

Previous literature has offered partial explanations for the benefits of FuzzMask. Papp et al. [14], [21] claimed that the advantage of FuzzMask lies in modeling PVE in PET images, and Pham et al. [15] argued that the fuzzy segmentation simplifies the handling of spatial‑information variables at boundaries. However, they did not fully elaborate on the underlying mechanism. Our study clarified that the superiority of FuzzMask stems from two mechanisms: the equalization of signal intensities across uncertainty regions and the gradient-weighted statistical modeling of peritumoral microenvironment.

The equalization of intensities across uncertainty regions enables RFs insensitive to the variations caused by contour position fluctuation. In H&N tumors, particularly OPC and NPC, the peritumoral region often exhibits significant intensity fluctuations in CT due to adjacent bone, soft tissue, and air cavities. Consequently, contour variations can introduce extreme intensity artifacts that compromise feature reliability. Our results (Fig. 3(a)) indicated that both shrinking (i.e., ShrMask) and expanding (i.e., ExtMask) the BinMask can improve the reliability of RF. Nevertheless, these methods either discard peripheral information or introduce noise. The FuzzMask method addressed this by implementing a gradient transition that focuses on the core while weighing peritumoral intensities, effectively balancing the exclusion of extreme fluctuations with the retention of boundary information and achieving intensity equalization. The Venn analysis (category (1110) in Fig. 3(b)) confirmed that the improvements across three masks were concentrated in Intensity- and GLCM-derived features. This is consistent with previous research [30] suggesting that fuzzy logic imparts a smoothing effect on intensity histograms, thereby reducing susceptibility to noise. Furthermore, GLCM visualizations in Fig. S3 of the Supplementary Material confirmed that FuzzMask effectively suppressed the statistical impact of extreme high and low gray levels, thereby preventing interference from extreme intensities.

The superior performance of FuzzMask in relevance analysis highlights the utility of its fuzzy boundary band. Generated by a Gaussian filter, this boundary extended approximately 2–4 mm from the gross tumor volume (GTV), effectively modeling a “fuzzy” clinical target volume (CTV). From a clinical pathology perspective, this peritumoral zone is a heterogeneous tumor microenvironment (TME), characterized by a gradient of microscopic tumor cell infiltration and immune cell response. The weighted, gradual transition of FuzzMask mathematically simulated this natural infiltration gradient. This approach shows encouraging signs of modeling the underlying biology more effectively than the hard boundaries of BinMask. Furthermore, this weighted scheme transformed the values in intermediate matrices (e.g., GLCM) from discrete integers to continuous real numbers, enabling a more nuanced capture of peritumoral heterogeneity. This increased precision reduced inter-feature correlations, thereby preserving a higher number of independent feature clusters (Fig. 4). By potentially offering a better model of the tumor’s boundary, the FuzzMask thus extracted a more representative and less redundant feature set.

This study observed the decoupling of radiomic reliability and model predictive accuracy. Survival modeling with ExtMask outperformed BinMask, consistent with previous studies [14], [31], [32]. In contrast, despite yielding a more reliable feature pool (Fig. 3) and more reliable predictions (Fig. 5(c)), the application of FuzzMask offered only marginal, non-significant gains in C-index and AUC (Fig. 5(a), (b)). This outcome, however, underscores a critical distinction: predictive power and reliability are independent and equally important metrics. From a clinical translation perspective, a model with modest but highly reproducible predictive power is arguably more valuable than a marginally more accurate but unstable one [8]. By improving the ICC of model predictions, the proposed FuzzMask directly addressed a core challenge for the clinical adoption of radiomics: the trustworthiness and consistency of its outputs.

This study is limited by its retrospective design and the use of heterogeneous public datasets with varying scanners and acquisition protocols, which highlight the need for prospective, multi-center validation. Furthermore, while repeating the modeling process 50 times provided a practical balance between computational efficiency and statistical stability, employing a higher number of iterations (e.g., 100 or more) could theoretically offer a more rigorous assessment of model variance. Although the “GTV + fuzzy CTV” model achieved only modest predictive improvement, this likely reflects the complexity of interpreting the rich information captured rather than a lack of prognostic potential. Elucidating the biological basis of features derived from the fuzzy CTV region will be essential for developing advanced models that fully leverage TME interactions.

In conclusion, the FuzzMask technique is a powerful tool for improving the reliability and robustness of CT-based radiomics in H&N cancer. By addressing the critical issue of delineation uncertainty, it strengthens the foundation for clinical translation, shifting the focus from pursuing marginal gains in accuracy to ensuring the delivery of consistent and trustworthy results.

## CRediT authorship contribution statement

**Jin Cao:** Writing – review & editing, Writing – original draft, Validation, Methodology, Investigation, Conceptualization. **Jiang Zhang:** Writing – review & editing, Methodology. **Xinzhi Teng:** Writing – review & editing, Methodology. **Xinyu Zhang:** Writing – review & editing, Methodology. **Saikit Lam:** Writing – review & editing, Methodology. **Ta Zhou:** Writing – review & editing, Methodology, Conceptualization. **Yuanpeng Zhang:** Writing – review & editing, Methodology. **Jing Cai:** Writing – review & editing, Methodology, Conceptualization.

## Declaration of competing interest

The authors declare that they have no known competing financial interests or personal relationships that could have appeared to influence the work reported in this paper.

## Footnotes

1 https://github.com/JinCAO-PolyU/Fuzzy-radiomics-feature.

## References

[1] Lambin P., Rios-Velazquez E., Leijenaar R., Carvalho S., Stiphout R.G., Granton P. (2012). Radiomics: extracting more information from medical images using advanced feature analysis. Eur J Cancer.

[2] Neher P., Hirjak D., Maier-Hein K. (2024). Radiomic tractometry reveals tract-specific imaging biomarkers in white matter. Nat Commun.

[3] Ruan B., Chen M., Zhuang Q., Guan L., Xie W., Wang L. (2025). Chest computed tomography–based radiomics for the diagnosis and prognosis of pulmonary hypertension. J Am Heart Assoc.

[4] Zhang Y.P., Zhang X.Y., Cheng Y.T., Li B., Teng X.Z., Zhang J. (2023). Artificial intelligence-driven radiomics study in cancer: the role of feature engineering and modeling. Mil Med Res.

[5] Zhang X., Teng X., Zhang J., Lai Q., Cai J. (2024). Enhancing pathological complete response prediction in breast cancer: the role of dynamic characterization of DCE-MRI and its association with tumor heterogeneity. Breast Cancer Res.

[6] Yip S., Aerts H. (2016). Applications and limitations of radiomics. Phys Med Biol.

[7] Gillies R., Kinahan P., Hricak H. (2016). Radiomics: images are more than pictures, they are data. Radiology.

[8] Zhao B. (2021). Understanding sources of variation to improve the reproducibility of radiomics. Front Oncol.

[9] Jaarsma-Coes M.G., Klaassen L., Vu T.K., Klaver Y.L., Rodrigues M.F., Nabarro C. (2023). Inter-Observer variability in MR-based target volume delineation of uveal melanoma. Adv Radiat Oncol.

[10] Willemink M.J., Mastrodicasa D., Madani M.H., Codari M., Chepelev L.L., Mistelbauer G. (2023). Inter-observer variability of expert-derived morphologic risk predictors in aortic dissection. Eur Radiol.

[11] Mitra S. (2021). Deep learning with radiogenomics towards personalized management of gliomas. IEEE Rev Biomed Eng.

[12] Scalco E., Rizzo G., Mastropietro A. (2022). The stability of oncologic MRI radiomic features and the potential role of deep learning: a review. Phys Med Biol.

[13] Wei H., Zheng T., Zhang X., Wu Y., Chen Y., Zheng C. (2024). MRI radiomics based on deep learning automated segmentation to predict early recurrence of hepatocellular carcinoma. Insights Imaging.

[14] Grahovac M., Spielvogel C.P., Krajnc D., Ecsedi B., Weidinger T.T., Rasul S. (2023). Machine learning predictive performance evaluation of conventional and fuzzy radiomics in clinical cancer imaging cohorts. Eur J Nucl Med Mol I.

[15] Pham D.L., Prince J.L. (1999). Adaptive fuzzy segmentation of magnetic resonance images. IEEE T Med Imaging.

[16] Huang Y., Wang T., Basanta H. (2020). Using fuzzy mask R-CNN model to automatically identify tomato ripeness. IEEE Access.

[17] Solaiman B., Fiset R., Cavayas F. (1998). Automatic road extraction using fuzzy mask concepts. Sens Manag Environ.

[18] Chen H., Chen H., Sun C.H., Wang W. (2024). Apply fuzzy mask to improve monocular depth estimation. Int J Fuzzy Syst.

[19] Altman D. (1994). Fuzzy set theoretic approaches for handling imprecision in spatial analysis. Int J Geogr Inf Syst.

[20] Bjørke J.T. (2004). Topological relations between fuzzy regions: derivation of verbal terms. Fuzzy Set Syst.

[21] Papp L., Rausch I., Hacker M., Beyer T. (2019). Fuzzy radiomics: a novel approach to minimize the effects of target delineation on radiomic models. Nuklearmedizin.

[22] Welch M.L., Kim S., Hope A.J., Huang S.H., Lu Z., Marsilla J. (2024). RADCURE: an open-source head and neck cancer CT dataset for clinical radiation therapy insights. Med Phys.

[23] Zhang J. Radiotherapy data analysis and reporting (RADAR) toolkit: an end-to-end artificial intelligence development solution for precision medicine. https://theses.lib.polyu.edu.hk/handle/200/12551; 2023 [Accessed 25 Aug 2025].

[24] Xue C., Yuan J., Lo G., Chang A.T., Poon D.M., Wong O.L. (2021). Radiomics feature reliability assessed by intraclass correlation coefficient: a systematic review. Quant Imag Med Surg.

[25] Shrout P.E., Fleiss J.L. (1979). Intraclass correlations: uses in assessing rater reliability. Psychol Bull.

[26] Zhang J., Lam S., Teng X., Ma Z., Han X., Zhang Y. (2023). Radiomic feature repeatability and its impact on prognostic model generalizability: a multi-institutional study on nasopharyngeal carcinoma patients. Radiother Oncol.

[27] Koo T.K., Li M.Y. (2016). A guideline of selecting and reporting intraclass correlation coefficients for reliability research. J Chiropr Med.

[28] Teng X., Zhang J., Zwanenburg A., Sun J., Huang Y., Lam S. (2022). Building reliable radiomic models using image perturbation. Sci Rep.

[29] Teng X., Zhang J., Ma Z., Zhang Y., Lam S., Li W. (2022). Improving radiomic model reliability using robust features from perturbations for head-and-neck carcinoma. Front Oncol.

[30] Jawahar C.V., Ray A.K. (1996). Fuzzy statistics of digital images. IEEE Signal Proc Let.

[31] Zeng C., Zhang W., Liu M., Liu J., Zheng Q., Li J. (2023). Efficacy of radiomics model based on the concept of gross tumor volume and clinical target volume in predicting occult lymph node metastasis in non-small cell lung cancer. Front Oncol.

[32] Huang Y.C., Huang S.M., Yeh J.H., Chang T.C., Tsan D.L., Lin C.Y. (2024). Utility of CT radiomics and delta radiomics for survival evaluation in locally advanced nasopharyngeal carcinoma with concurrent chemoradiotherapy. Diagnostics.

