# Supplementary material for: Fuzzy masks: boosting radiomic reliability in head and neck tumors amid delineation uncertainty
Source: Phys Imaging Radiat Oncol. 2026 Mar 15;38:100947. doi: 10.1016/j.phro.2026.100947 (PMC13018959; doi:10.1016/j.phro.2026.100947)
Supplement: Supplementary Data 1 [file mmc1.pdf]

Supplementary Materials for Fuzzy Masks: Boosting Radiomic Reliability in Head and Neck Tumors Amid Delineation Uncertainty

Jin Cao, Jiang Zhang, Xinzhi Teng, Xinyu Zhang, Saikit Lam, Ta Zhou, Yuanpeng Zhang, Jing Cai

A. Generation of shrunk binary mask and extended binary mask from fuzzy mask

According to the theory of fuzzy region, the generated fuzzy mask (FuzzMask) and medical image can be denoted as a fuzzy tumor region $\mathcal{T}$:

$$\mathcal{T} = \{I(x,y), \mu_{\mathcal{T}}(x,y) | (x,y) \in I\} \quad (1)$$

Where I denotes the medical image, (x,y) is the position in I, and $\mu_{\mathcal{T}}(x,y)$ is the membership value which describes the degree of $I(x,y)$ belongs to tumor region. And the FuzzMask U becomes $\{\mu_{\mathcal{T}}(x,y) | (x,y) \in I\}$. Therefore, the generation process of the FuzzMask is the process of determining $\mu_{\mathcal{T}}(x,y)$. According to fuzzy set theory, membership value can be calculated through membership function or specified based on prior knowledge. Considering that pixels are mostly discrete values, this study opts for the latter approach.

To examine the impact of boundary fuzzification, the shrunk binary mask (ShrMask) and extended binary mask (ExtMask) were also generated for comparison. The shrunk region S can be generated as follows by utilizing the α-cut technique in fuzzy region:

$$S = \mathcal{T}_{\alpha=1} = \{I(x,y) | \mu_{\mathcal{T}}(x,y) \geq 1\} \quad (2)$$

This indicates that each position in the shrunk mask belongs to the tumor region with a degree of 1 (*i.e.*, 100%). The extended region E, which refers to the region where each position with a membership value greater than 0, was defined as the support of the fuzzy region $\mathcal{T}$ according to fuzzy region theory as follows:

$$E = supp(\mathcal{T}) = \{I(x,y) | \mu_{\mathcal{T}}(x,y) > 0\} \quad (3)$$

As a result, the ShrMask is a binary mask which describes the positions with the membership value of 1 in FuzzMask, and the ExtMask is a binary mask which describes the positions with the membership value greater than 0 in FuzzMask.

B. The preprocessing of medical image and mask

The calculation procedures for shape and intensity features are different with texture features when using FuzzMask. Morphological features are derived from the contour, and first-order statistical features are computed based on intensities within the contour, regardless of the mask values. Therefore, a clear tumor contour remains essential. In this context, a binarized FuzzMask is generated by the α-cut of the fuzzy boundary ∂T of the fuzzy region T within the FuzzMask, where α is set to 0.5 [1]. Consequently, the calculation process for shape and intensity features follows a traditional approach akin to that of a smoothed binary mask, while texture features are computed directly utilizing the preprocessed FuzzMask (**Figure S1**).

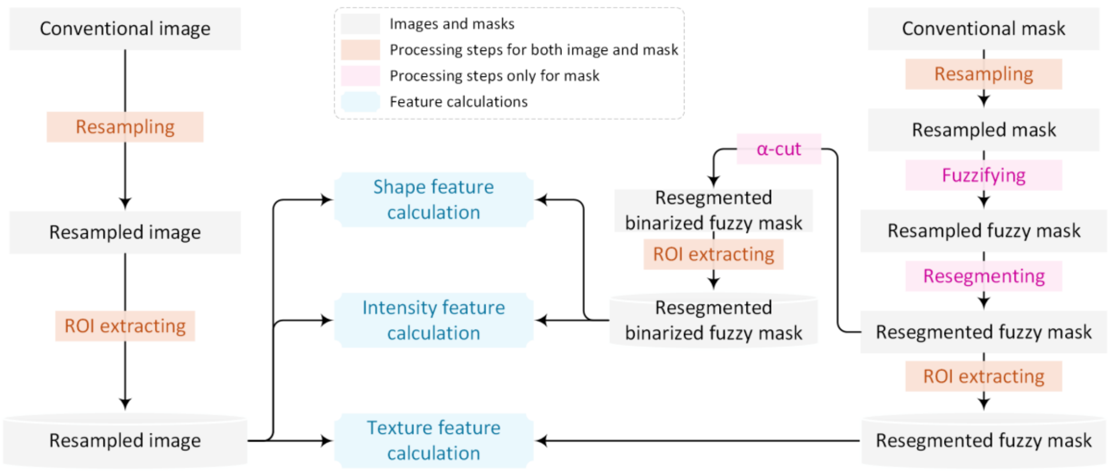

Figure S1. The preprocessing procedures of medical image and mask for calculating radiomic features.

C. Specific extraction example of radiomic texture features using FuzzMask

The distinction in texture feature calculation processes between using a FuzzMask and a BinMask lies in the substitution of the statistical counting method of intermediate matrices with the averaging of mask values. For example, when filling the intermediate matrix of Gray-level Co-occurrence Matrix (GLCM), considering the image in **Figure S2(a)** and the first BinMask in **Figure S2(b)** as a reference, $P(g = 3, h = 2)$ equals 2, denoting the count of neighboring pixel pairs with values 3 and 2 from left to right within the mask. When collating the average mask values of neighboring pixels according to the formula in **Figure S2(c)**, $P(g = 3, h = 2)$ remains at 2. However, this value shifts to 1.6 when utilizing FuzzMask (as depicted in **Figure S2(d)**. Therefore, this method is applicable to both conventional binary masks as a traditional statistical process and fuzzy masks as an extension of the statistical process. This approach is not limited to the statistical process of GLCM but can also be extended to the other five intermediate matrices.

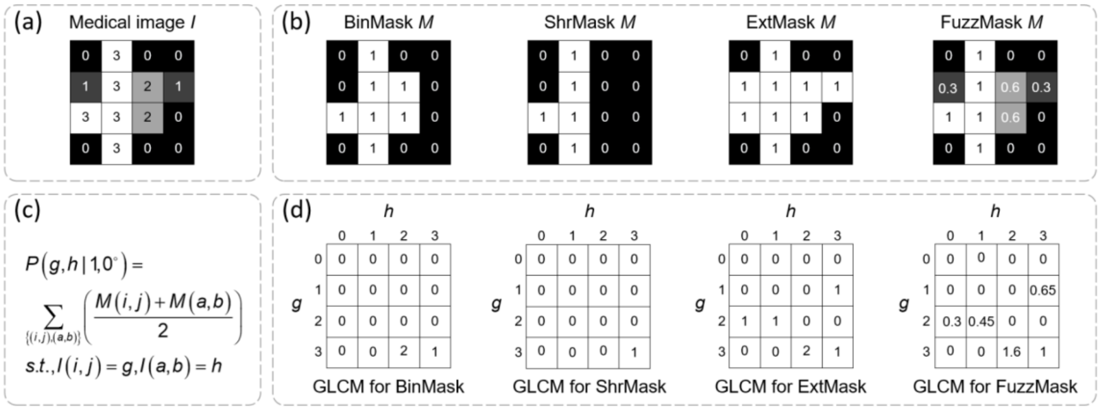

Figure S2. Illustration of differences in GLCMs generated using four different masks. (a) A 2D image *I* consists of 4 discrete gray levels (0-3). (b) The masks of BinMask, ShrMask, ExtMask, and FuzzMask. (c) The accumulation rule of GLCM, defined as the average of mask values when considering only left-to-right neighbors (i.e., g⟶h) in image. (d) The filled GLCMs derived from the four masks.

D. Parameter settings of perturbation process

Table S1. The parameter value sets of perturbations. The parameter values of each perturbation are randomly selected from the value sets.

Parameters	Value sets
Rotation angles	[-10, 0, 10]
Translation distances	[0, 0.4, 0.8]
Additive noise	[0, 1, 2, 5]
Contour randomization	[5, 10]

E. Visualization of filled intermediate matrices using FuzzMask

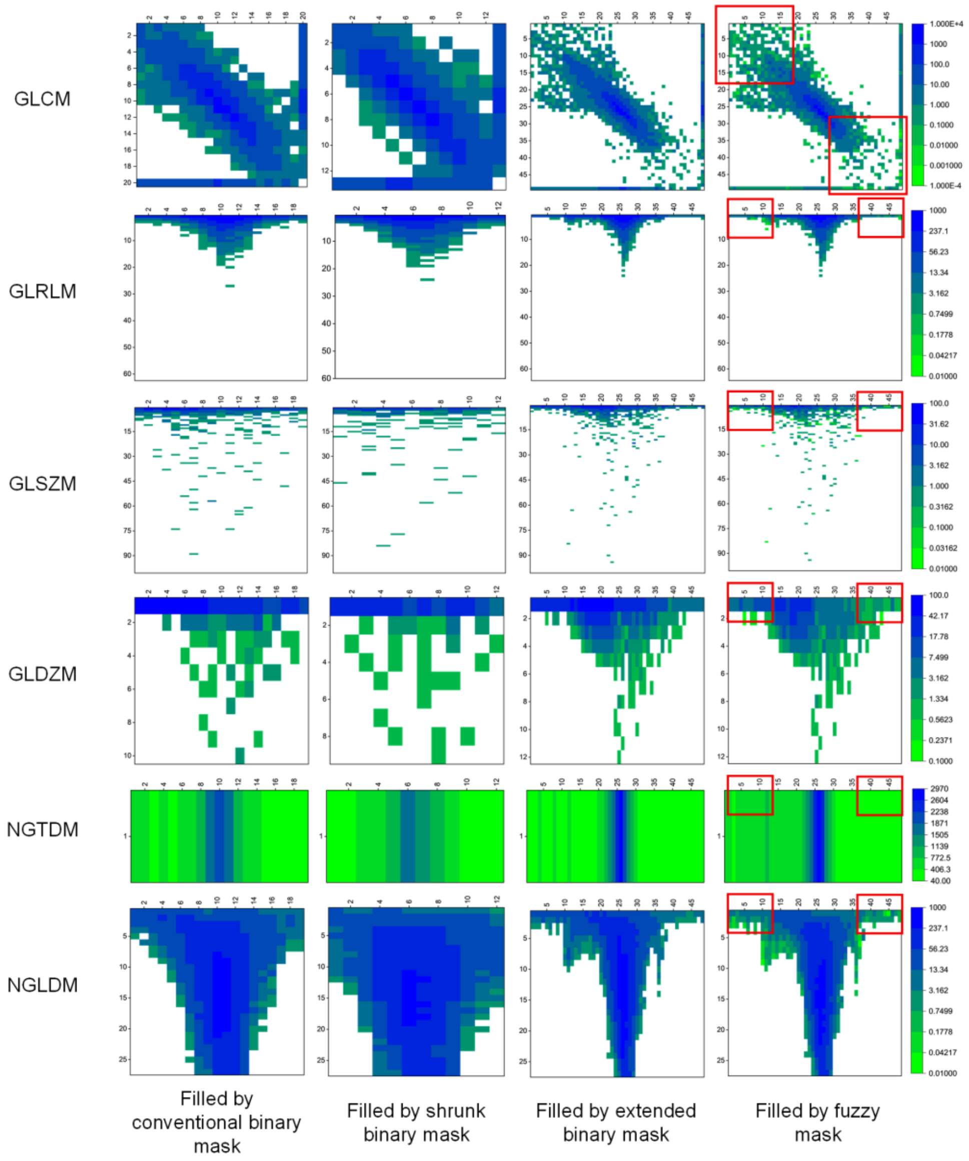

Figure S3. The visualization of filled intermediate matrices across four comparative masks.

F. Venn data for three cohorts

Table S2. Venn data for laryngeal cancer cohort.

Category	Feature names
0001	None
0010	'Texture_ngldm_DependenceCountPercentage3D'
0011	None
0100	'Intensity_IntensityVariance3D'<br>'Intensity_IntensityInterquartileRange3D'<br>'Intensity_IntensityRange3D'<br>'Intensity_MeanAbsoluteDeviation3D'<br>'Intensity_RobustMeanAbsoluteDeviation3D'<br>'Intensity_MeanDiscretisedIntensity3D'<br>'Intensity_DiscretisedIntensityVariance3D'<br>'Intensity_MedianDiscretisedIntensity3D'<br>'Intensity_TenthDiscretisedIntensityPercentile3D'<br>'Intensity_MaximumDiscretisedIntensity3D'<br>'Intensity_IntensityHistogramMode3D'<br>'Intensity_DiscretisedIntensityInterquartileRange3D'<br>'Intensity_DiscretisedIntensityRange3D'<br>'Intensity_IntensityHistogramMeanAbsoluteDeviation3D'<br>'Intensity_IntensityHistogramRobustMeanAbsoluteDeviation3D'<br>'Intensity_MaximumHistogramGradientIntensity3D'<br>'Intensity_MinimumHistogramGradientIntensity3D'<br>'Intensity_IntensityFractionDifference3D'<br>'Texture_glcm_JointAverageAveraged3D'<br>'Texture_glcm_JointVarianceAveraged3D'<br>'Texture_glcm_SumAverageAveraged3D'<br>'Texture_glcm_SumVarianceAveraged3D'<br>'Texture_glcm_AutoCorrelationAveraged3D'<br>'Texture_glcm_ClusterTendencyAveraged3D'<br>'Texture_glcm_ClusterShadeAveraged3D'<br>'Texture_glcm_ClusterProminenceAveraged3D'<br>'Texture_glcm_JointAverageMerged3D'<br>'Texture_glcm_JointVarianceMerged3D'<br>'Texture_glcm_SumAverageMerged3D'<br>'Texture_glcm_SumVarianceMerged3D'<br>'Texture_glcm_AutoCorrelationMerged3D'<br>'Texture_glcm_ClusterTendencyMerged3D'<br>'Texture_glcm_ClusterShadeMerged3D'<br>'Texture_glcm_ClusterProminenceMerged3D'<br>'Texture_glrlm_HighGrayLevelRunEmphasisAveraged3D'<br>'Texture_glrlm_ShortRunHighGrayLevelEmphasisAveraged3D'<br>'Texture_glrlm_LongRunHighGrayLevelEmphasisAveraged3D'<br>'Texture_glrlm_GrayLevelVarianceAveraged3D'<br>'Texture_glrlm_HighGrayLevelRunEmphasisMerged3D'<br>'Texture_glrlm_ShortRunHighGrayLevelEmphasisMerged3D'<br>'Texture_glrlm_LongRunHighGrayLevelEmphasisMerged3D'<br>'Texture_glrlm_GrayLevelVarianceMerged3D'<br>'Texture_glszm_HighGrayLevelZoneEmphasis3D'<br>'Texture_glszm_SmallZoneHighGrayLevelEmphasis3D'<br>'Texture_glszm_GrayLevelVariance3D'<br>'Texture_gldzm_HighGrayLevelZoneDistanceEmphasis3D'<br>'Texture_gldzm_GrayLevelDistanceVariance3D'<br>'Texture_ngldm_HighGrayLevelCountEmphasis3D'<br>'Texture_ngldm_GrayLevelDependenceVariance3D'
0101	None
0110	'Texture_gldzm_SmallDistanceEmphasis3D'<br>'Texture_gldzm_NormalisedZoneDistanceNonUniformity3D'<br>'Texture_gldzm_ZoneDistanceEntropy3D'
0111	'Texture_glszm_ZoneSizeEntropy3D'<br>'Texture_gldzm_LargeDistanceHighGrayLevelEmphasis3D'
1000	'Intensity_TenPercentIntensityFraction3D'<br>'Texture_glcm_NormalisedInverseDifferenceMomentAveraged3D'

	'Texture_glcm_NormalisedInverseDifferenceMomentMerged3D'<br>'Texture_glszm_SmallZoneEmphasis3D'<br>'Texture_glszm_NormalisedZoneSizeNonUniformity3D'
1001	None
1010	'Texture_glcm_InformationCorrelation1Averaged3D'<br>'Texture_glcm_InformationCorrelation2Averaged3D'<br>'Texture_glszm_LargeZoneLowGrayLevelEmphasis3D'
1011	'Texture_ngtdm_Busyness3D'
1100	'Intensity_IntensityKurtosis3D'<br>'Intensity_MedianAbsoluteDeviation3D'<br>'Intensity_NinetiethDiscretisedIntensityPercentile3D'<br>'Intensity_IntensityHistogramMedianAbsoluteDeviation3D'<br>'Texture_glcm_DifferenceVarianceAveraged3D'<br>'Texture_glcm_ContrastAveraged3D'<br>'Texture_glcm_DifferenceVarianceMerged3D'<br>'Texture_glcm_ContrastMerged3D'<br>'Texture_glrlm_RunEntropyAveraged3D'<br>'Texture_glrlm_RunEntropyMerged3D'
1101	None
1110	'Intensity_DiscretisedIntensityKurtosis3D'<br>'Texture_glcm_DifferenceAverageAveraged3D'<br>'Texture_glcm_DissimilarityAveraged3D'<br>'Texture_glcm_InverseVarianceAveraged3D'<br>'Texture_glcm_DifferenceAverageMerged3D'<br>'Texture_glcm_DissimilarityMerged3D'<br>'Texture_glcm_InverseVarianceMerged3D'<br>'Texture_glcm_InformationCorrelation2Merged3D'<br>'Texture_gldzm_SmallDistanceHighGrayLevelEmphasis3D'<br>'Texture_ngtdm_Contrast3D'<br>'Texture_ngtdm_Complexity3D'<br>'Texture_ngtdm_Strength3D'<br>'Texture_ngldm_LowDependenceHighGrayLevelEmphasis3D'<br>'Texture_ngldm_HighDependenceHighGrayLevelEmphasi3D'
1111	'Shape_VolumeVoxelCount3D'<br>'Shape_SurfaceAreaMesh3D'<br>'Shape_SurfaceVolumeRatio3D'<br>'Shape_Compactness1_3D'<br>'Shape_Compactness2_3D'<br>'Shape_SphericalDisproportion3D'<br>'Shape_Sphericity3D'<br>'Shape_Asphericity3D'<br>'Shape_Maximum3dDiameter3D'<br>'Shape_MajorAxisLength3D'<br>'Shape_MinorAxisLength3D'<br>'Shape_LeastAxisLength3D'<br>'Shape_Elongation3D'<br>'Shape_Flatness3D'<br>'Shape_VolumeDensityAABB_3D'<br>'Shape_AreaDensityAABB_3D'<br>'Shape_VolumeDensityAEE_3D'<br>'Shape_AreaDensityAEE_3D'<br>'Shape_VolumeDensityConvexHull3D'<br>'Shape_AreaDensityConvexHull3D'<br>'Shape_IntegratedIntensity3D'<br>'Intensity_LocalIntensityPeak3D'<br>'Intensity_GlobalIntensityPeak3D'<br>'Intensity_MeanIntensity3D'<br>'Intensity_MedianIntensity3D'<br>'Intensity_MinimumIntensity3D'<br>'Intensity_TenthIntensityPercentile3D'<br>'Intensity_NinetiethIntensityPercentile3D'<br>'Intensity_MaximumIntensity3D'<br>'Intensity_IntensityEnergy3D'<br>'Intensity_RootMeanSquare3D'<br>'Intensity_DiscretisedIntensityEntropy3D'<br>'Intensity_DiscretisedIntensityUniformity3D'<br>'Intensity_MaximumHistogramGradient3D'

	'Intensity_MinimumHistogramGradient3D'<br>'Texture_glcm_JointMaximumAveraged3D'<br>'Texture_glcm_JointEntropyAveraged3D'<br>'Texture_glcm_DifferenceEntropyAveraged3D'<br>'Texture_glcm_SumEntropyAveraged3D'<br>'Texture_glcm_AngularSecondMomentAveraged3D'<br>'Texture_glcm_InverseDifferenceAveraged3D'<br>'Texture_glcm_NormalisedInverseDifferenceAveraged3D'<br>'Texture_glcm_InverseDifferenceMomentAveraged3D'<br>'Texture_glcm_JointMaximumMerged3D'<br>'Texture_glcm_JointEntropyMerged3D'<br>'Texture_glcm_DifferenceEntropyMerged3D'<br>'Texture_glcm_SumEntropyMerged3D'<br>'Texture_glcm_AngularSecondMomentMerged3D'<br>'Texture_glcm_InverseDifferenceMerged3D'<br>'Texture_glcm_NormalisedInverseDifferenceMerged3D'<br>'Texture_glcm_InverseDifferenceMomentMerged3D'<br>'Texture_glcm_InformationCorrelation1Merged3D'<br>'Texture_glrlm_ShortRunsEmphasisAveraged3D'<br>'Texture_glrlm_LongRunsEmphasisAveraged3D'<br>'Texture_glrlm_GrayLevelNonUniformityAveraged3D'<br>'Texture_glrlm_NormalisedGrayLevelNonUniformityAveraged3D'<br>'Texture_glrlm_RunLengthNonUniformityAveraged3D'<br>'Texture_glrlm_NormalisedRunLengthNonUniformityAveraged3D'<br>'Texture_glrlm_RunPercentageAveraged3D'<br>'Texture_glrlm_RunLengthVarianceAveraged3D'<br>'Texture_glrlm_ShortRunsEmphasisMerged3D'<br>'Texture_glrlm_LongRunsEmphasisMerged3D'<br>'Texture_glrlm_GrayLevelNonUniformityMerged3D'<br>'Texture_glrlm_NormalisedGrayLevelNonUniformityMerged3D'<br>'Texture_glrlm_RunLengthNonUniformityMerged3D'<br>'Texture_glrlm_NormalisedRunLengthNonUniformityMerged3D'<br>'Texture_glrlm_RunPercentageMerged3D'<br>'Texture_glrlm_RunLengthVarianceMerged3D'<br>'Texture_glszm_LargeZoneEmphasis3D'<br>'Texture_glszm_LargeZoneHighGrayLevelEmphasis3D'<br>'Texture_glszm_GrayLevelNonUniformity3D'<br>'Texture_glszm_ZoneSizeNonUniformity3D'<br>'Texture_glszm_ZonePercentage3D'<br>'Texture_glszm_ZoneSizeVariance3D'<br>'Texture_gldzm_LargeDistanceEmphasis3D'<br>'Texture_gldzm_GrayLevelDistanceNonUniformity3D'<br>'Texture_gldzm_ZoneDistanceNonUniformity3D'<br>'Texture_gldzm_ZoneDistancePercentage3D'<br>'Texture_gldzm_ZoneDistanceVariance3D'<br>'Texture_ngtdm_Coarseness3D'<br>'Texture_ngldm_LowDependenceEmphasis3D'<br>'Texture_ngldm_HighDependenceEmphasis3D'<br>'Texture_ngldm_GrayLevelDependenceNonUniformity3D'<br>'Texture_ngldm_NormalisedGrayLevelDependenceNonUniformity3D'<br>'Texture_ngldm_DependenceCountNonUniformity3D'<br>'Texture_ngldm_NormalisedDependenceCountNonUniformity3D'<br>'Texture_ngldm_DependenceCountVariance3D'<br>'Texture_ngldm_DependenceCountEntropy3D'<br>'Texture_ngldm_DependenceCountEnergy3D'

Table S3. Venn data for oropharyngeal cancer cohort

**Category**	**Feature names**
0001	None
0010	'Texture_ngldm_DependenceCountPercentage3D'
0011	None
0100	'Texture_glszm_LowGrayLevelZoneEmphasis3D'<br>'Texture_gldzm_LowGrayLevelZoneDistanceEmphasis3D'<br>'Texture_gldzm_SmallDistanceLowGrayLevelEmphasis3D'
0101	'Texture_ngldm_LowDependenceLowGrayLevelEmphasis3D'

0110	None
0111	None
1000	'Texture_glszm_SmallZoneEmphasis3D'
1001	None
1010	'Intensity_IntensityHistogramCoeffcientOfVariation3D'<br>'Intensity_TenPercentIntensityFraction3D'<br>'Texture_glszm_LargeZoneLowGrayLevelEmphasis3D'<br>'Texture_ngtdm_Contrast3D'<br>'Texture_ngtdm_Strength3D'
1011	None
1100	'Intensity_IntensitySkewness3D'<br>'Intensity_IntensityKurtosis3D'<br>'Intensity_IntensityFractionDifference3D'<br>'Texture_glszm_NormalisedGrayLevelNonUniformity3D'<br>'Texture_glszm_NormalisedZoneSizeNonUniformity3D'<br>'Texture_gldzm_NormalisedGrayLevelDistanceNonUniformity3D'
1101	None
1110	'Intensity_MeanDiscretisedIntensity3D'<br>'Intensity_DiscretisedIntensitySkewness3D'<br>'Intensity_DiscretisedIntensityKurtosis3D'<br>'Intensity_MedianDiscretisedIntensity3D'<br>'Intensity_TenthDiscretisedIntensityPercentile3D'<br>'Intensity_IntensityHistogramMode3D'<br>'Intensity_MaximumHistogramGradientIntensity3D'<br>'Intensity_MinimumHistogramGradientIntensity3D'<br>'Texture_glcm_JointAverageAveraged3D'<br>'Texture_glcm_SumAverageAveraged3D'<br>'Texture_glcm_AutoCorrelationAveraged3D'<br>'Texture_glcm_JointAverageMerged3D'<br>'Texture_glcm_SumAverageMerged3D'<br>'Texture_glcm_AutoCorrelationMerged3D'<br>'Texture_glrlm_HighGrayLevelRunEmphasisAveraged3D'<br>'Texture_glrlm_ShortRunHighGrayLevelEmphasisAveraged3D'<br>'Texture_glrlm_LongRunHighGrayLevelEmphasisAveraged3D'<br>'Texture_glrlm_HighGrayLevelRunEmphasisMerged3D'<br>'Texture_glrlm_ShortRunHighGrayLevelEmphasisMerged3D'<br>'Texture_glrlm_LongRunHighGrayLevelEmphasisMerged3D'<br>'Texture_gldzm_SmallDistanceHighGrayLevelEmphasis3D'<br>'Texture_ngtdm_Busyness3D'<br>'Texture_ngldm_HighGrayLevelCountEmphasis3D'<br>'Texture_ngldm_HighDependenceHighGrayLevelEmphasi3D'
1111	'Shape_VolumeVoxelCount3D'<br>'Shape_SurfaceAreaMesh3D'<br>'Shape_SurfaceVolumeRatio3D'<br>'Shape_Compactness1_3D'<br>'Shape_Compactness2_3D'<br>'Shape_SphericalDisproportion3D'<br>'Shape_Sphericity3D'<br>'Shape_Asphericity3D'<br>'Shape_Maximum3dDiameter3D'<br>'Shape_MajorAxisLength3D'<br>'Shape_MinorAxisLength3D'<br>'Shape_LeastAxisLength3D'<br>'Shape_Elongation3D'<br>'Shape_Flatness3D'<br>'Shape_VolumeDensityAABB_3D'<br>'Shape_AreaDensityAABB_3D'<br>'Shape_VolumeDensityAEE_3D'<br>'Shape_AreaDensityAEE_3D'<br>'Shape_VolumeDensityConvexHull3D'<br>'Shape_AreaDensityConvexHull3D'<br>'Shape_IntegratedIntensity3D'<br>'Intensity_LocalIntensityPeak3D'<br>'Intensity_GlobalIntensityPeak3D'<br>'Intensity_MeanIntensity3D'<br>'Intensity_IntensityVariance3D'

	'Intensity_MedianIntensity3D'<br>'Intensity_MinimumIntensity3D'<br>'Intensity_TenthIntensityPercentile3D'<br>'Intensity_NinetiethIntensityPercentile3D'<br>'Intensity_MaximumIntensity3D'<br>'Intensity_IntensityInterquartileRange3D'<br>'Intensity_IntensityRange3D'<br>'Intensity_MeanAbsoluteDeviation3D'<br>'Intensity_RobustMeanAbsoluteDeviation3D'<br>'Intensity_MedianAbsoluteDeviation3D'<br>'Intensity_IntensityEnergy3D'<br>'Intensity_RootMeanSquare3D'<br>'Intensity_DiscretisedIntensityVariance3D'<br>'Intensity_NinetiethDiscretisedIntensityPercentile3D'<br>'Intensity_MaximumDiscretisedIntensity3D'<br>'Intensity_DiscretisedIntensityInterquartileRange3D'<br>'Intensity_DiscretisedIntensityRange3D'<br>'Intensity_IntensityHistogramMeanAbsoluteDeviation3D'<br>'Intensity_IntensityHistogramRobustMeanAbsoluteDeviation3D'<br>'Intensity_IntensityHistogramMedianAbsoluteDeviation3D'<br>'Intensity_DiscretisedIntensityEntropy3D'<br>'Intensity_DiscretisedIntensityUniformity3D'<br>'Intensity_MaximumHistogramGradient3D'<br>'Intensity_MinimumHistogramGradient3D'<br>'Texture_glcm_JointMaximumAveraged3D'<br>'Texture_glcm_JointVarianceAveraged3D'<br>'Texture_glcm_JointEntropyAveraged3D'<br>'Texture_glcm_DifferenceAverageAveraged3D'<br>'Texture_glcm_DifferenceVarianceAveraged3D'<br>'Texture_glcm_DifferenceEntropyAveraged3D'<br>'Texture_glcm_SumVarianceAveraged3D'<br>'Texture_glcm_SumEntropyAveraged3D'<br>'Texture_glcm_AngularSecondMomentAveraged3D'<br>'Texture_glcm_ContrastAveraged3D'<br>'Texture_glcm_DissimilarityAveraged3D'<br>'Texture_glcm_InverseDifferenceAveraged3D'<br>'Texture_glcm_NormalisedInverseDifferenceAveraged3D'<br>'Texture_glcm_InverseDifferenceMomentAveraged3D'<br>'Texture_glcm_NormalisedInverseDifferenceMomentAveraged3D'<br>'Texture_glcm_InverseVarianceAveraged3D'<br>'Texture_glcm_CorrelationAveraged3D'<br>'Texture_glcm_ClusterTendencyAveraged3D'<br>'Texture_glcm_ClusterShadeAveraged3D'<br>'Texture_glcm_ClusterProminenceAveraged3D'<br>'Texture_glcm_InformationCorrelation1Averaged3D'<br>'Texture_glcm_InformationCorrelation2Averaged3D'<br>'Texture_glcm_JointMaximumMerged3D'<br>'Texture_glcm_JointVarianceMerged3D'<br>'Texture_glcm_JointEntropyMerged3D'<br>'Texture_glcm_DifferenceAverageMerged3D'<br>'Texture_glcm_DifferenceVarianceMerged3D'<br>'Texture_glcm_DifferenceEntropyMerged3D'<br>'Texture_glcm_SumVarianceMerged3D'<br>'Texture_glcm_SumEntropyMerged3D'<br>'Texture_glcm_AngularSecondMomentMerged3D'<br>'Texture_glcm_ContrastMerged3D'<br>'Texture_glcm_DissimilarityMerged3D'<br>'Texture_glcm_InverseDifferenceMerged3D'<br>'Texture_glcm_NormalisedInverseDifferenceMerged3D'<br>'Texture_glcm_InverseDifferenceMomentMerged3D'<br>'Texture_glcm_NormalisedInverseDifferenceMomentMerged3D'<br>'Texture_glcm_InverseVarianceMerged3D'<br>'Texture_glcm_CorrelationMerged3D'<br>'Texture_glcm_ClusterTendencyMerged3D'<br>'Texture_glcm_ClusterShadeMerged3D'<br>'Texture_glcm_ClusterProminenceMerged3D'<br>'Texture_glcm_InformationCorrelation1Merged3D'

	'Texture_glcm_InformationCorrelation2Merged3D'<br>'Texture_glrlm_ShortRunsEmphasisAveraged3D'<br>'Texture_glrlm_LongRunsEmphasisAveraged3D'<br>'Texture_glrlm_GrayLevelNonUniformityAveraged3D'<br>'Texture_glrlm_NormalisedGrayLevelNonUniformityAveraged3D'<br>'Texture_glrlm_RunLengthNonUniformityAveraged3D'<br>'Texture_glrlm_NormalisedRunLengthNonUniformityAveraged3D'<br>'Texture_glrlm_RunPercentageAveraged3D'<br>'Texture_glrlm_GrayLevelVarianceAveraged3D'<br>'Texture_glrlm_RunLengthVarianceAveraged3D'<br>'Texture_glrlm_RunEntropyAveraged3D'<br>'Texture_glrlm_ShortRunsEmphasisMerged3D'<br>'Texture_glrlm_LongRunsEmphasisMerged3D'<br>'Texture_glrlm_GrayLevelNonUniformityMerged3D'<br>'Texture_glrlm_NormalisedGrayLevelNonUniformityMerged3D'<br>'Texture_glrlm_RunLengthNonUniformityMerged3D'<br>'Texture_glrlm_NormalisedRunLengthNonUniformityMerged3D'<br>'Texture_glrlm_RunPercentageMerged3D'<br>'Texture_glrlm_GrayLevelVarianceMerged3D'<br>'Texture_glrlm_RunLengthVarianceMerged3D'<br>'Texture_glrlm_RunEntropyMerged3D'<br>'Texture_glszm_LargeZoneEmphasis3D'<br>'Texture_glszm_HighGrayLevelZoneEmphasis3D'<br>'Texture_glszm_SmallZoneHighGrayLevelEmphasis3D'<br>'Texture_glszm_LargeZoneHighGrayLevelEmphasis3D'<br>'Texture_glszm_GrayLevelNonUniformity3D'<br>'Texture_glszm_ZoneSizeNonUniformity3D'<br>'Texture_glszm_ZonePercentage3D'<br>'Texture_glszm_GrayLevelVariance3D'<br>'Texture_glszm_ZoneSizeVariance3D'<br>'Texture_glszm_ZoneSizeEntropy3D'<br>'Texture_gldzm_SmallDistanceEmphasis3D'<br>'Texture_gldzm_LargeDistanceEmphasis3D'<br>'Texture_gldzm_HighGrayLevelZoneDistanceEmphasis3D'<br>'Texture_gldzm_LargeDistanceHighGrayLevelEmphasis3D'<br>'Texture_gldzm_GrayLevelDistanceNonUniformity3D'<br>'Texture_gldzm_ZoneDistanceNonUniformity3D'<br>'Texture_gldzm_NormalisedZoneDistanceNonUniformity3D'<br>'Texture_gldzm_ZoneDistancePercentage3D'<br>'Texture_gldzm_GrayLevelDistanceVariance3D'<br>'Texture_gldzm_ZoneDistanceVariance3D'<br>'Texture_gldzm_ZoneDistanceEntropy3D'<br>'Texture_ngtdm_Coarseness3D'<br>'Texture_ngtdm_Complexity3D'<br>'Texture_ngldm_LowDependenceEmphasis3D'<br>'Texture_ngldm_HighDependenceEmphasis3D'<br>'Texture_ngldm_LowDependenceHighGrayLevelEmphasis3D'<br>'Texture_ngldm_GrayLevelDependenceNonUniformity3D'<br>'Texture_ngldm_NormalisedGrayLevelDependenceNonUniformity3D'<br>'Texture_ngldm_DependenceCountNonUniformity3D'<br>'Texture_ngldm_NormalisedDependenceCountNonUniformity3D'<br>'Texture_ngldm_GrayLevelDependenceVariance3D'<br>'Texture_ngldm_DependenceCountVariance3D'<br>'Texture_ngldm_DependenceCountEntropy3D'<br>'Texture_ngldm_DependenceCountEnergy3D'

Table S4. Venn data for nasopharyngeal carcinoma cohort

**Category**	**Feature names**
0001	None
0010	'Texture_ngldm_DependenceCountPercentage3D'
0011	'Texture_gldzm_LargeDistanceLowGrayLevelEmphasis3D'
0100	'Shape_CentreOfMassShift3D'<br>'Intensity_CoefficientOfVariation3D'<br>'Intensity_QuartileCoefficientOfDispersion3D'<br>'Texture_ngldm_LowDependenceLowGrayLevelEmphasis3D'

0101	'Intensity_TenthDiscretisedIntensityPercentile3D'
0110	None
0111	'Texture_glcm_ClusterShadeAveraged3D'<br>'Texture_glcm_ClusterShadeMerged3D'
1000	'Intensity_IntensitySkewness3D'<br>'Intensity_IntensityKurtosis3D'<br>'Intensity_DiscretisedIntensitySkewness3D'
1001	None
1010	'Intensity_DiscretisedIntensityKurtosis3D'
1011	'Intensity_IntensityHistogramCoeffcientOfVariation3D'<br>'Intensity_IntensityHistogramQuartileCoeffcientOfDispersion3D'<br>'Intensity_IntensityFractionDifference3D'<br>'Texture_glszm_LargeZoneLowGrayLevelEmphasis3D'
1100	None
1101	None
1110	'Texture_glszm_NormalisedGrayLevelNonUniformity3D'<br>'Texture_gldzm_NormalisedGrayLevelDistanceNonUniformity3D'<br>'Texture_ngtdm_Contrast3D'<br>'Texture_ngldm_HighDependenceHighGrayLevelEmphasi3D'
1111	'Shape_VolumeVoxelCount3D'<br>'Shape_SurfaceAreaMesh3D'<br>'Shape_SurfaceVolumeRatio3D'<br>'Shape_Compactness1_3D'<br>'Shape_Compactness2_3D'<br>'Shape_SphericalDisproportion3D'<br>'Shape_Sphericity3D'<br>'Shape_Asphericity3D'<br>'Shape_Maximum3dDiameter3D'<br>'Shape_MajorAxisLength3D'<br>'Shape_MinorAxisLength3D'<br>'Shape_LeastAxisLength3D'<br>'Shape_Elongation3D'<br>'Shape_Flatness3D'<br>'Shape_VolumeDensityAABB_3D'<br>'Shape_AreaDensityAABB_3D'<br>'Shape_VolumeDensityAEE_3D'<br>'Shape_AreaDensityAEE_3D'<br>'Shape_VolumeDensityConvexHull3D'<br>'Shape_AreaDensityConvexHull3D'<br>'Shape_IntegratedIntensity3D'<br>'Intensity_LocalIntensityPeak3D'<br>'Intensity_GlobalIntensityPeak3D'<br>'Intensity_MeanIntensity3D'<br>'Intensity_IntensityVariance3D'<br>'Intensity_MedianIntensity3D'<br>'Intensity_MinimumIntensity3D'<br>'Intensity_TenthIntensityPercentile3D'<br>'Intensity_NinetiethIntensityPercentile3D'<br>'Intensity_MaximumIntensity3D'<br>'Intensity_IntensityInterquartileRange3D'<br>'Intensity_IntensityRange3D'<br>'Intensity_MeanAbsoluteDeviation3D'<br>'Intensity_RobustMeanAbsoluteDeviation3D'<br>'Intensity_MedianAbsoluteDeviation3D'<br>'Intensity_IntensityEnergy3D'<br>'Intensity_RootMeanSquare3D'<br>'Intensity_MeanDiscretisedIntensity3D'<br>'Intensity_DiscretisedIntensityVariance3D'<br>'Intensity_MedianDiscretisedIntensity3D'<br>'Intensity_NinetiethDiscretisedIntensityPercentile3D'<br>'Intensity_MaximumDiscretisedIntensity3D'<br>'Intensity_IntensityHistogramMode3D'<br>'Intensity_DiscretisedIntensityInterquartileRange3D'<br>'Intensity_DiscretisedIntensityRange3D'<br>'Intensity_IntensityHistogramMeanAbsoluteDeviation3D'<br>'Intensity_IntensityHistogramRobustMeanAbsoluteDeviation3D'

	'Intensity_IntensityHistogramMedianAbsoluteDeviation3D'<br>'Intensity_DiscretisedIntensityEntropy3D'<br>'Intensity_DiscretisedIntensityUniformity3D'<br>'Intensity_MaximumHistogramGradient3D'<br>'Intensity_MaximumHistogramGradientIntensity3D'<br>'Intensity_MinimumHistogramGradient3D'<br>'Intensity_MinimumHistogramGradientIntensity3D'<br>'Texture_glcm_JointMaximumAveraged3D'<br>'Texture_glcm_JointAverageAveraged3D'<br>'Texture_glcm_JointVarianceAveraged3D'<br>'Texture_glcm_JointEntropyAveraged3D'<br>'Texture_glcm_DifferenceAverageAveraged3D'<br>'Texture_glcm_DifferenceVarianceAveraged3D'<br>'Texture_glcm_DifferenceEntropyAveraged3D'<br>'Texture_glcm_SumAverageAveraged3D'<br>'Texture_glcm_SumVarianceAveraged3D'<br>'Texture_glcm_SumEntropyAveraged3D'<br>'Texture_glcm_AngularSecondMomentAveraged3D'<br>'Texture_glcm_ContrastAveraged3D'<br>'Texture_glcm_DissimilarityAveraged3D'<br>'Texture_glcm_InverseDifferenceAveraged3D'<br>'Texture_glcm_NormalisedInverseDifferenceAveraged3D'<br>'Texture_glcm_InverseDifferenceMomentAveraged3D'<br>'Texture_glcm_NormalisedInverseDifferenceMomentAveraged3D'<br>'Texture_glcm_InverseVarianceAveraged3D'<br>'Texture_glcm_CorrelationAveraged3D'<br>'Texture_glcm_AutoCorrelationAveraged3D'<br>'Texture_glcm_ClusterTendencyAveraged3D'<br>'Texture_glcm_ClusterProminenceAveraged3D'<br>'Texture_glcm_InformationCorrelation1Averaged3D'<br>'Texture_glcm_InformationCorrelation2Averaged3D'<br>'Texture_glcm_JointMaximumMerged3D'<br>'Texture_glcm_JointAverageMerged3D'<br>'Texture_glcm_JointVarianceMerged3D'<br>'Texture_glcm_JointEntropyMerged3D'<br>'Texture_glcm_DifferenceAverageMerged3D'<br>'Texture_glcm_DifferenceVarianceMerged3D'<br>'Texture_glcm_DifferenceEntropyMerged3D'<br>'Texture_glcm_SumAverageMerged3D'<br>'Texture_glcm_SumVarianceMerged3D'<br>'Texture_glcm_SumEntropyMerged3D'<br>'Texture_glcm_AngularSecondMomentMerged3D'<br>'Texture_glcm_ContrastMerged3D'<br>'Texture_glcm_DissimilarityMerged3D'<br>'Texture_glcm_InverseDifferenceMerged3D'<br>'Texture_glcm_NormalisedInverseDifferenceMerged3D'<br>'Texture_glcm_InverseDifferenceMomentMerged3D'<br>'Texture_glcm_NormalisedInverseDifferenceMomentMerged3D'<br>'Texture_glcm_InverseVarianceMerged3D'<br>'Texture_glcm_CorrelationMerged3D'<br>'Texture_glcm_AutoCorrelationMerged3D'<br>'Texture_glcm_ClusterTendencyMerged3D'<br>'Texture_glcm_ClusterProminenceMerged3D'<br>'Texture_glcm_InformationCorrelation1Merged3D'<br>'Texture_glcm_InformationCorrelation2Merged3D'<br>'Texture_glrlm_ShortRunsEmphasisAveraged3D'<br>'Texture_glrlm_LongRunsEmphasisAveraged3D'<br>'Texture_glrlm_HighGrayLevelRunEmphasisAveraged3D'<br>'Texture_glrlm_ShortRunHighGrayLevelEmphasisAveraged3D'<br>'Texture_glrlm_LongRunHighGrayLevelEmphasisAveraged3D'<br>'Texture_glrlm_GrayLevelNonUniformityAveraged3D'<br>'Texture_glrlm_NormalisedGrayLevelNonUniformityAveraged3D'<br>'Texture_glrlm_RunLengthNonUniformityAveraged3D'<br>'Texture_glrlm_NormalisedRunLengthNonUniformityAveraged3D'<br>'Texture_glrlm_RunPercentageAveraged3D'<br>'Texture_glrlm_GrayLevelVarianceAveraged3D'<br>'Texture_glrlm_RunLengthVarianceAveraged3D'

	'Texture_glrlm_RunEntropyAveraged3D'<br>'Texture_glrlm_ShortRunsEmphasisMerged3D'<br>'Texture_glrlm_LongRunsEmphasisMerged3D'<br>'Texture_glrlm_HighGrayLevelRunEmphasisMerged3D'<br>'Texture_glrlm_ShortRunHighGrayLevelEmphasisMerged3D'<br>'Texture_glrlm_LongRunHighGrayLevelEmphasisMerged3D'<br>'Texture_glrlm_GrayLevelNonUniformityMerged3D'<br>'Texture_glrlm_NormalisedGrayLevelNonUniformityMerged3D'<br>'Texture_glrlm_RunLengthNonUniformityMerged3D'<br>'Texture_glrlm_NormalisedRunLengthNonUniformityMerged3D'<br>'Texture_glrlm_RunPercentageMerged3D'<br>'Texture_glrlm_GrayLevelVarianceMerged3D'<br>'Texture_glrlm_RunLengthVarianceMerged3D'<br>'Texture_glrlm_RunEntropyMerged3D'<br>'Texture_glszm_SmallZoneEmphasis3D'<br>'Texture_glszm_LargeZoneEmphasis3D'<br>'Texture_glszm_HighGrayLevelZoneEmphasis3D'<br>'Texture_glszm_SmallZoneHighGrayLevelEmphasis3D'<br>'Texture_glszm_LargeZoneHighGrayLevelEmphasis3D'<br>'Texture_glszm_GrayLevelNonUniformity3D'<br>'Texture_glszm_ZoneSizeNonUniformity3D'<br>'Texture_glszm_NormalisedZoneSizeNonUniformity3D'<br>'Texture_glszm_ZonePercentage3D'<br>'Texture_glszm_GrayLevelVariance3D'<br>'Texture_glszm_ZoneSizeVariance3D'<br>'Texture_glszm_ZoneSizeEntropy3D'<br>'Texture_gldzm_SmallDistanceEmphasis3D'<br>'Texture_gldzm_LargeDistanceEmphasis3D'<br>'Texture_gldzm_HighGrayLevelZoneDistanceEmphasis3D'<br>'Texture_gldzm_SmallDistanceHighGrayLevelEmphasis3D'<br>'Texture_gldzm_LargeDistanceHighGrayLevelEmphasis3D'<br>'Texture_gldzm_GrayLevelDistanceNonUniformity3D'<br>'Texture_gldzm_ZoneDistanceNonUniformity3D'<br>'Texture_gldzm_NormalisedZoneDistanceNonUniformity3D'<br>'Texture_gldzm_ZoneDistancePercentage3D'<br>'Texture_gldzm_GrayLevelDistanceVariance3D'<br>'Texture_gldzm_ZoneDistanceVariance3D'<br>'Texture_gldzm_ZoneDistanceEntropy3D'<br>'Texture_ngtdm_Coarseness3D'<br>'Texture_ngtdm_Busyness3D'<br>'Texture_ngtdm_Complexity3D'<br>'Texture_ngtdm_Strength3D'<br>'Texture_ngldm_LowDependenceEmphasis3D'<br>'Texture_ngldm_HighDependenceEmphasis3D'<br>'Texture_ngldm_HighGrayLevelCountEmphasis3D'<br>'Texture_ngldm_LowDependenceHighGrayLevelEmphasis3D'<br>'Texture_ngldm_GrayLevelDependenceNonUniformity3D'<br>'Texture_ngldm_NormalisedGrayLevelDependenceNonUniformity3D'<br>'Texture_ngldm_DependenceCountNonUniformity3D'<br>'Texture_ngldm_NormalisedDependenceCountNonUniformity3D'<br>'Texture_ngldm_GrayLevelDependenceVariance3D'<br>'Texture_ngldm_DependenceCountVariance3D'<br>'Texture_ngldm_DependenceCountEntropy3D'<br>'Texture_ngldm_DependenceCountEnergy3D'

Reference

[1] Bjørke, JT. Topological relations between fuzzy regions: derivation of verbal terms. Fuzzy Sets Syst 2024; 141(3): 449-467. https://doi.org/10.1016/S0165-0114(02)00574-2.

[2] Grahovac M, Spielvogel CP, Krajnc D, Ecsedi B, Weidinger TT, Rasul S, et al. Machine learning predictive performance evaluation of conventional and fuzzy radiomics in clinical cancer imaging cohorts. Eur J Nucl Med Mol Imaging 2023; 50(6): 1607-1620. https://doi.org/10.1007/s00259-023-06127-1.
